# Impact of the COVID-19 pandemic on diagnosis, stage, and initial treatment of breast cancer in the Netherlands: a population-based study

**DOI:** 10.1186/s13045-021-01073-7

**Published:** 2021-04-17

**Authors:** Anouk H. Eijkelboom, Linda de Munck, Marie-Jeanne T. F. D. Vrancken Peeters, Mireille J. M. Broeders, Luc J. A. Strobbe, Monique E. M. M. Bos, Marjanka K. Schmidt, Cristina Guerrero Paez, Marjolein L. Smidt, Maud Bessems, Janneke Verloop, Sabine Linn, Marc B. I. Lobbes, Aafke H. Honkoop, Desirée H. J. G. van den Bongard, Pieter J. Westenend, Jelle Wesseling, C. Willemien Menke-van der Houven van Oordt, Vivianne C. G. Tjan-Heijnen, Sabine Siesling, the NABON COVID-19 Consortium and the COVID and Cancer-NL Consortium, Jolanda C. van Hoeve, Matthias A. W. Merkx, Niek J. de Wit, Irene Dingemans, Iris D. Nagtegaal, A. Wilbrink, Carla H. van Gils, Henk C. P. M. van Weert, Marcel Verheij, Ernest J. T. Luiten, A. Elise van Leeuwen-Stok, Agnes Jager, Linetta B. Koppert, Maartje J. Hooning, Liesbeth J. Boersma, Carolien P. Schröder, Helena M. Verkooijen, Quirine C. van Rossum-Schornagel, Susanne van der Velde, Eveliene Manten-Horst, Nicolien T. van Ravensteyn, Joke C. Korevaar, Ester J. M. Siemerink, Thijs van Dalen, Annette W. G. van der Velden, Marc A. M. Mureau

**Affiliations:** 1 Department of Research and Development, Netherlands Comprehensive Cancer Organisation (IKNL), Godebaldkwartier 419, 3511 DT Utrecht, The Netherlands; 2 Department of Surgery, Netherlands Cancer Institute–Antoni van Leeuwenhoek Hospital, Plesmanlaan 121, 1066 CX Amsterdam, The Netherlands; 3 Department of Surgery, Amsterdam UMC, Meibergdreef 9, 1105 AZ Amsterdam, The Netherlands; 4 Department for Health Evidence, Radboud University Medical Center, Geert Grooteplein Zuid 10, 6525 GA Nijmegen, The Netherlands; 5 Dutch Expert Centre for Screening, Wijchenseweg 101, 6538 SW Nijmegen, The Netherlands; 6 Department of Surgical Oncology, Canisius Wilhelmina Hospital, Weg door Jonkerbos 100, 6532 SZ Nijmegen, The Netherlands; 7 Department of Medical Oncology, Erasmus Medical Centre Cancer Institute, Doctor Molewaterplein 40, 3015 GD Rotterdam, The Netherlands; 8 Division of Molecular Pathology, Netherlands Cancer Institute–Antoni van Leeuwenhoek Hospital, Plesmanlaan 121, 1066 CX Amsterdam, The Netherlands; 9 Dutch Breast Cancer Society (BVN), Godebaldkwartier 363, 3511 DT Utrecht, The Netherlands; 10 Department of Surgery, Maastricht University Medical Centre, P. Debyelaan 25, 6229 HX Maastricht, the Netherlands; 11 GROW School for Oncology and Development Biology, Maastricht University, Univeristeitssingel 40, 6220 ER Maastricht, the Netherlands; 12 Department of Surgery, Jeroen Bosch Hospital, Henri Dunantstraat 1, 5223 GZ ’s-Hertogenbosch, The Netherlands; 13 Division of Diagnostic Oncology and Molecular Pathology, Netherlands Cancer Institute–Antoni van Leeuwenhoek Hospital, Plesmanlaan 121, 1066 CX Amsterdam, The Netherlands; 14 Department of Pathology, University Medical Center Utrecht, Heidelberglaan 100, 3584 CX Utrecht, The Netherlands; 15 Department of Medical Imaging, Zuyderland Medical Center, Sittard-Geleen, Dr. H. van der Hoffplein 1, 6162 BG Geleen, The Netherlands; 16 Department of Radiology and Nuclear Medicine, Maastricht University Medical Center, P. Debyelaan 25, 6229 HX Maastricht, The Netherlands; 17 Department of Medical Oncology, Isala Clinics, Dokter van Heesweg 2, 8025 AB Zwolle, The Netherlands; 18 Department of Radiation Oncology, Amsterdam UMC, Meibergdreef 9, 1105 AZ Amsterdam, The Netherlands; 19 Laboratory of Pathology, Karel Lotsyweg 145, 3318 AL Dordrecht, The Netherlands; 20 Department of Pathology, Leiden University Medical Center, Albinusdreef 2, 2333 ZA Leiden, The Netherlands; 21 Department of Medical Oncology, Cancer Center Amsterdam, Amsterdam UMC, Location Vrije Universiteit Medical Centre, De Boelelaan 1117, 1081 HV Amsterdam, The Netherlands; 22 Department of Medical Oncology, Maastricht University Medical Centre, P. Debyelaan 25, 6229 HX Maastricht, The Netherlands; 23 Department of Health Technology and Services Research, Technical Medical Centre, University of Twente, Drienerlolaan 5, 7522 NB Enschede, The Netherlands

**Keywords:** COVID-19, Breast cancer, Incidence, Screening, Treatment, Stage, Population-based

## Abstract

**Background:**

The onset of the COVID-19 pandemic forced the Dutch national screening program to a halt and increased the burden on health care services, necessitating the introduction of specific breast cancer treatment recommendations from week 12 of 2020. We aimed to investigate the impact of COVID-19 on the diagnosis, stage and initial treatment of breast cancer.

**Methods:**

Women included in the Netherlands Cancer Registry and diagnosed during four periods in weeks 2–17 of 2020 were compared with reference data from 2018/2019 (averaged). Weekly incidence was calculated by age group and tumor stage. The number of women receiving initial treatment within 3 months of diagnosis was calculated by period, initial treatment, age, and stage. Initial treatment, stratified by tumor behavior (ductal carcinoma in situ [DCIS] or invasive), was analyzed by logistic regression and adjusted for age, socioeconomic status, stage, subtype, and region. Factors influencing time to treatment were analyzed by Cox regression.

**Results:**

Incidence declined across all age groups and tumor stages (except stage IV) from 2018/2019 to 2020, particularly for DCIS and stage I disease (*p* < 0.05). DCIS was less likely to be treated within 3 months (odds ratio [OR]_wks2–8_: 2.04, OR_wks9–11_: 2.18). Invasive tumors were less likely to be treated initially by mastectomy with immediate reconstruction (OR_wks12–13_: 0.52) or by breast conserving surgery (OR_wks14–17_: 0.75). Chemotherapy was less likely for tumors diagnosed in the beginning of the study period (OR_wks9–11_: 0.59, OR_wks12–13_: 0.66), but more likely for those diagnosed at the end (OR_wks14–17_: 1.31). Primary hormonal treatment was more common (OR_wks2–8_: 1.23, OR_wks9–11_: 1.92, OR_wks12–13_: 3.01). Only women diagnosed in weeks 2–8 of 2020 experienced treatment delays.

**Conclusion:**

The incidence of breast cancer fell in early 2020, and treatment approaches adapted rapidly. Clarification is needed on how this has affected stage migration and outcomes.

**Supplementary Information:**

The online version contains supplementary material available at 10.1186/s13045-021-01073-7.

## Background

The COVID-19 pandemic still has the world in its grip. In the Netherlands, the first COVID-19 cases were identified at the end of February 2020 in the south of the country [1]. The virus then spread gradually across the country, affecting mainly southern and central regions. From the second week of March, societal measures were introduced, such as social distancing, closing schools, and urging people to stay at home as much as possible. A key driver of this policy was to protect the most vulnerable in society, such as the elderly and the chronically ill. In addition to having wide reaching effects on society, health care services switched focus to patients with COVID-19, thereby generating pressures on all other health care domains. Efforts were made to ease the burden on health care services overwhelmed by the surge of COVID-19 patients, to reallocate personal protective equipment to health care staff tackling COVID-19, and to mitigate the spread of COVID-19.

The Dutch breast cancer screening program invites women aged 50–74 years for biennial screening mammography. However, in line with efforts to prioritize care for COVID-19, national screening programs were halted from March 16, 2020 (week 12), including that for breast cancer. Combined with the decreased number of referrals from general practitioners (GPs) [2], this has led to a drop in the number of breast cancer diagnoses in the Netherlands [3]. To accommodate the many changes brought about by COVID-19, the Dutch Society of Medical Oncology (NVMO), the Dutch Society of Surgical Oncology (NVCO), and the Dutch Society of Radiotherapy and Oncology (NVRO) collaborated to issue recommended treatment strategies to be applicable from week 12 (Additional file 1: Table 1) [4]. These recommendations had three goals: 1) to continue safe and effective oncological care for all new and known patients; 2) to decrease the risk of infection for patients and staff; and 3) to ensure the availability of protective materials, staff, and intensive care unit capacity for critically ill patients with COVID-19.

In April 2020, the multidisciplinary National Breast Cancer Organization of the Netherlands (NABON) established its *NABON COVID-19 Consortium* to monitor, evaluate, and learn from the impact of COVID-19 on breast cancer care. This is the first study performed by this consortium and seeks to report on the impact of the COVID-19 pandemic on the incidence of breast cancer and the initial treatment for patients within 3 months after diagnosis.

## Methods

### Patients

Women older than 18 years and diagnosed with breast cancer during weeks 2–17 of 2018, 2019, or 2020 were selected for inclusion from the Netherlands Cancer Registry (NCR). The NCR is hosted by the Netherlands Comprehensive Cancer Organization (IKNL) and has records of all newly diagnosed malignancies notified by the Nationwide Histopathology and Cytopathology Data Network and Archive (PALGA) since 1989. Within 3 months after breast cancer diagnosis, the following data are gathered: patient characteristics (e.g., age at diagnosis, gender, socioeconomic status [SES]), detection method (e.g., screening), tumor characteristics (e.g., TNM stage, morphology, grade, and hormone receptor status), and primary treatment (e.g., surgery, neoadjuvant therapy, and systemic therapy). Data collection is completed 9 months after diagnosis by including further details on adjuvant and other treatments.

The present study was approved by the Privacy Review Board of the NCR and data were made available until week 17, 2020. We analyzed data on the diagnosis and initial treatment (i.e. within the first 3 months after diagnosis) of breast cancer patients.

### Definitions

Weeks 2–17 of 2020 were considered the “COVID-19 period” and were divided into four: period A to cover weeks 2–8 (i.e., before the COVID-19 pandemic); period B, weeks 9–11 (i.e., between the first COVID-19 case and the social lockdown); period C, weeks 12–13 (i.e., the lockdown was introduced and screening was halted); and period D, weeks 14–17 (i.e., referrals from the screening program ended). We included averaged data for weeks 2–17 of 2018 and 2019 as a reference.

Tumors were grouped by their method of detection (screening or symptomatic presentation). Age at diagnosis was grouped into ages < 40, 40–49, 50–64, 65–74, and > 74 years. SES was determined based on the patient’s postal code at the time of diagnosis. Hormone receptor (HR) and human epidermal growth factor receptor 2 (HER2) status were combined in the variable “tumor subtype,” as follows: HR+/HER2+, HR+/HER2−, HR−/HER2+, and HR−/HER2−. Stage (ductal carcinoma in situ [DCIS] and stage I, II, II, IV) was defined according to the TNM staging system [5]. Patients were also categorized into one of the five regions (provinces) in the Netherlands where they were diagnosed: the north (Groningen, Friesland, Drenthe), the middle east (Overijssel, Flevoland), the middle (Gelderland, Utrecht), the west (Noord-Holland, Zuid-Holland, Zeeland), and the south (Limburg, Noord-Brabant).

### Statistical analysis

All data were analyzed using Stata version 16.1 software (StataCorp, College Station, Texas, USA). The baseline characteristics of participants diagnosed in weeks 2–17 of either 2018/2019 or 2020 are presented descriptively. Chi-squared tests were used to compare patients diagnosed in periods A, B, C, and D of 2020 with those diagnosed in the same periods for 2018/2019. A two-sided *p* value < 0.05 was considered statistically significant. Incidence and treatment data were then compared in detail.

The incidence of newly diagnosed breast tumors was calculated for each week and expressed per 1 million women living in the Netherlands at the start of the year using data from Statistics Netherlands (CBS) [6]. To calculate the number of potentially missed breast cancers diagnoses, the incidence of DCIS and of invasive breast cancer in weeks 9–17 of 2020 was subtracted from the average incidence of DCIS and of invasive cancer in weeks 9–17 of 2018/2019. This was then divided by 1 million and multiplied by 8,759,554 (i.e., the number of women living in the Netherlands at the start of 2020). The mean decrease in incidence (min–max) per individual hospital in weeks 9–17 of 2020 was calculated as a percentage of the number of diagnoses in weeks 9–17 of 2018/2019.

The incidence of newly diagnosed screen-detected and non-screen-detected tumors, expressed per 1 million women aged 50–74 years living in the Netherlands at the start of the year, was determined for each week. The average incidence per week was calculated for periods A, B, C, and D of 2020 and for the corresponding weeks in 2018/2019 by age group, tumor stage, and region. Incidences were also expressed per 1 million women of a given age group living in the Netherlands at the start of the year. The incidence in each period of 2020 was compared with the average for the same period in 2018/2019 using STATA’s iri command with a midp-calculation [7]. A one-sided *p* value of < 0.05 was considered statistically significant because we expected a decline in breast cancer diagnoses. The average number of patients hospitalized with COVID-19 per week was calculated per period for each region to reflect the severity of the outbreak, using data from the National Institute for Public Health and Environment [1].

The number of women receiving an initial treatment within 3 months after diagnosis, out of the average number of women diagnosed per week, was calculated. Data were stratified by period (weeks 2–17 of 2018/2019, and periods A, B, C, and D of 2020) and type of initial treatment. Initial treatment included no treatment, breast conserving surgery (BCS), mastectomy with immediate breast reconstruction (IBR), mastectomy without IBR, chemotherapy (with or without targeted therapy), hormonal therapy, and other initial therapy (e.g., targeted therapy only, radiotherapy, and metastasis-directed radiotherapy). Data were further stratified by tumor stage and age group. Initial treatments during the four COVID-19 periods were compared with those given during the reference period using the Mantel–Haenszel test. Analyses were adjusted for tumor stage or age group, depending on the stratification variable. When heterogeneity was present, age groups and tumor stages were analyzed separately. A two-sided *p* value of < 0.05 was deemed statistically significant for these analyses.

Logistic regression was performed to calculate the odds ratios and 95% confidence intervals (CIs) for the association between diagnosis period and chance of receiving a certain treatment as initial treatment. Analyses were performed per initial treatment and tumor behavior (DCIS or invasive). For patients with invasive cancers, the association between diagnosis period and time (in days) from diagnosis to initial treatment was determined by Cox proportional hazard models estimating hazard ratios and 95%CIs. Analyses were performed per initial treatment, were only patients who received the treatment of interest were included. Both analyses were adjusted for age group, SES, region, subtype, and stage. A two-sided *p* value of < 0.05 was deemed statistically significant. No analysis was performed for delay in treatment for DCIS because this was recommended (Additional file 1: Table 1).

## Results

The characteristics of patients diagnosed in weeks 2–17 of 2018 and 2019 were comparable (Additional file 1: Table 2), with 5685 and 5838 patients diagnosed, respectively. By contrast, 4769 patients were diagnosed in the same period of 2020 (Table 1). The absolute number of breast cancer diagnoses decreased most prominently in period D for patients aged 50–64 or 65–74 years (*p* < 0.01); moreover, the absolute numbers of DCIS and stage I tumor diagnoses fell most in period D (*p* < 0.01).

**Table 1:** Baseline characteristics by diagnosis period

	Weeks 2–8			Weeks 9–11			Weeks 12–13			Weeks 14–17		
	2018/2019^a^	2020	*p*	2018/2019^a^	2020	*p*	2018/2019^a^	2020	*p*	2018/2019^a^	2020	*p*
Patients	2547.5	2692		1074.5	995		737.5	436		1402	646	
Age												
< 40	129.5 (5.1)	142 (5.3)	0.02	51 (4.8)	44 (4.4)	0.55	36.5 (5.0)	19 (4.4)	0.42	54 (3.9)	64 (9.9)	< 0.01
40–49	389.5 (15.3)	372 (13.8)		158.5 (14.8)	128 (12.9)		104.5 (14.2)	62 (14.2)		204.5 (14.6)	111 (17.2)	
50–64	912.5 (35.8)	898 (33.4)		387.5 (36.1)	368 (37.0)		266.5 (36.1)	175 (40.1)		524.5 (37.4)	200 (31.0)	
65–74	674 (26.5)	771 (28.6)		284.5 (26.5)	282 (28.3)		206.5 (28.0)	121 (27.8)		358 (25.5)	118 (18.3)	
> 74	442 (17.4)	509 (18.9)		193 (18.0)	173 (17.4)		123.5 (16.8)	59 (13.5)		261 (18.6)	153 (23.7)	
SES												
High	782.5 (30.7)	788 (29.3)	< 0.01	295.5 (27.5)	316 (31.8)	0.04	211.5 (28.7)	133 (30.5)	0.47	410.5 (29.3)	206 (31.9)	0.28
Medium	992.5 (39.0)	1169 (43.4)		421 (39.2)	379 (38.1)		282.5 (38.3)	153 (35.1)		569.5 (40.6)	242 (37.5)	
Low	763.5 (30.0)	719 (26.7)		355 (33.0)	298 (30.0)		239.5 (32.5)	148 (33.9)		413.5 (29.5)	193 (29.9)	
Unknown	9 (0.4)	16 (0.6)		3 (0.3)	2 (0.2)		4 (0.5)	2 (0.5)		8.5 (0.6)	5 (0.8)	
Morphology												
DCIS	309 (12.1)	352 (13.1)	0.44	119.5 (11.1)	125 (12.6)	0.33	79.5 (10.8)	65 (14.9)	0.03	178 (12.7)	42 (6.5)	0.00
Invasive ductal	1761.5 ( 69.2)	1828 (67.9)		756 (70.4)	671 (67.4)		504.5 (68.4)	294 (67.4)		951 (67.8)	480 (74.3)	
Invasive lobular	342.5 (13.4)	380 (14.1)		135 (12.6)	141 (14.2)		122 (16.5)	55 (12.6)		198 (14.1)	92 (14.2)	
Other	134.5 (5.3)	132 (4.9)		64 (6.0)	58 (5.8)		31.5 (4.3)	22 (5.1)		75 (5.4)	32 (5.0)	
Stage												
DCIS	309 (12.2)	352 (13.2)	0.25	119.5 (11.1)	125 (12.6)	0.69	79.5 (10.8)	65 (15.0)	0.09	178 (12.7)	42 (6.6)	< 0.01
Stage I	1044.5 (41.1)	1075 (40.4)		446 (41.6)	418 (42.2)		306 (41.6)	167 (38.6)		559.5 (40.0)	212 (33.1)	
Stage II	828.5 (32.6)	891 (33.5)		358 (33.4)	316 (31.9)		247 (33.6)	142 (32.8)		473.5 (33.8)	269 (42.0)	
Stage III	233.5 (9.2)	211 (7.9)		97 (9.0)	82 (8.3)		72 (9.8)	35 (8.1)		122 (8.7)	64 (10.0)	
Stage IV	128 (5.0)	134 (5.0)		52 (4.9)	49 (5.0)		31 (4.2)	24 (5.5)		66.5 (4.8)	54 (8.4)	
Subtype												
HR+/HER2+	210 (8.2)	171 (6.4)	0.01	74.5 (6.9)	64 (6.4)	0.88	48.5 (6.6)	30 (6.9)	0.59	111.5 (8.0)	62 (9.6)	0.12
HR+/HER2–	1678 (65.9)	1743 (64.8)		717 (66.7)	657 (66.0)		510 (69.2)	286 (65.6)		921.5 (65.7)	428 (66.3)	
HR−/HER2+	83 (3.3)	65 (2.4)		41.5 (3.9)	33 (3.3)		30 (4.1)	11 (2.5)		42 (3.0)	30 (4.6)	
HR−/HER2−	249 (9.8)	275 (10.2)		112 (10.4)	98 (9.9)		64.5 (8.8)	35 (8.0)		137.5 (9.8)	76 (11.8)	
Unknown	327.5 (12.9)	438 (16.3)		129.5 (12.1)	143 (14.4)		84.5 (11.5)	74 (17.0)		189.5 (13.5)	50 (7.7)	
Region												
North	257.5 (10.1)	254 (9.4)	0.02	111.5 (10.4)	99 (10.0)	0.74	74 (10.0)	46 (10.6)	0.17	135 (9.6)	71 (11.0)	0.86
Middle East	230.5 (9.1)	257 (9.6)		98.5 (9.2)	97 (9.8)		71.5 (9.7)	55 (12.6)		123.5 (8.8)	59 (9.1)	
Middle	488 (19.2)	589 (21.9)		214 (19.9)	208 (20.9)		139 (18.9)	94 (21.6)		297.5 (21.2)	132 (20.4)	
West	1010.5 (39.7)	991 (36.8)		406 (37.8)	384 (38.6)		304.5 (41.3)	165 (37.8)		538 (38.4)	246 (38.1)	
South	560.5 (22.0)	600 (22.3)		244.5 (22.8)	207 (20.8)		148.5 (20.1)	76 (17.4)		308 (22.0)	138 (21.4)	
Screened												
Yes	810 (31.8)	837 (31.1)	0.87	354.5 (33.0)	342 (34.4)	0.43	256.5 (34.8)	172 (39.5)	0.12	455.5 (32.5)	12 (1.9)	0.00
No	727.5 (28.6)	762 (28.3)		299 (27.8)	275 (27.6)		201.5 (27.3)	107 (24.5)		403.5 (28.8)	282 (43.7)	
Not eligible	961 (37.7)	1023 (38.0)		402.5 (37.5)	345 (34.7)		264.5 (35.9)	140 (32.1)		519.5 (37.1)	328 (50.8)	
Unknown	49 (1.9)	70 (2.6)		18.5 (1.7)	33 (3.3)		15 (2.0)	17 (3.9)		23.5 (1.7)	24 (3.7)	

Data are reported as N (%) of patients diagnosed in weeks 2–17 of 2018/2019 or 2020

The *p* value was calculated on known values only, using the chi-square test to compare patients diagnosed in weeks 2–8, 9–11, 12–13, or 14–17 of 2020 with those diagnosed in the same period from the average for the years 2018 and 2019

DCIS, ductal carcinoma in situ; HER2, human epidermal growth factor receptor 2; HR, hormone receptor; SES, socioeconomic status

^a^The average was taken over 2018 and 2019

### Incidence

**Fig. 1:**
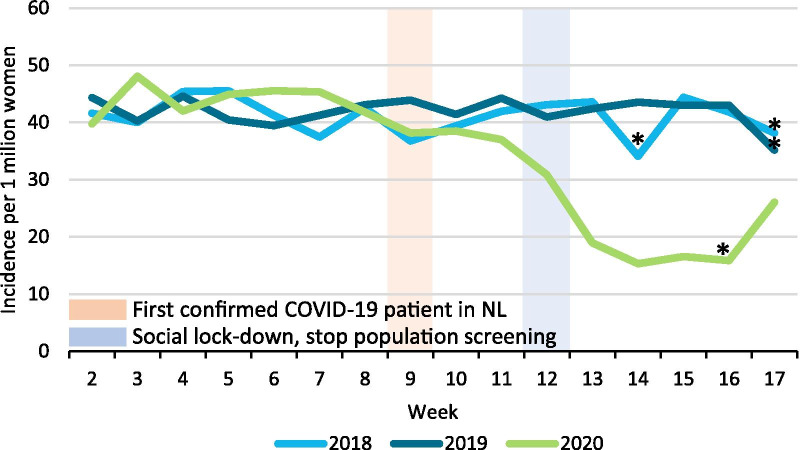
Incidence of breast cancer per week. Incidence is expressed per 1 million women living in the Netherlands at the start of the year. *The week only includes four working days due to public holidays

**Fig. 2:**
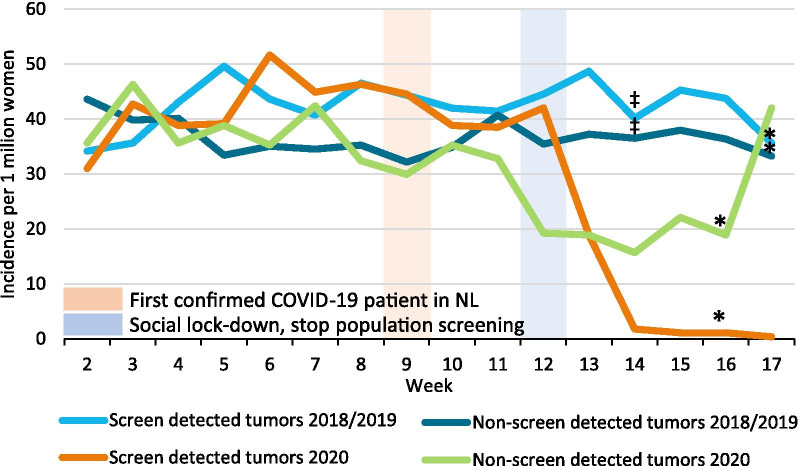
Incidence of screen- and non-screen-detected tumors per week. Incidence is expressed per 1 million women aged 50–74 years living in the Netherlands at the start of the year. *The week only includes four working days due to public holidays. ^‡^The week only includes on average four and a half working days due to public holidays in 2018

The incidence of breast cancer started to decline from week 9, with an even steeper decline from week 11. Breast cancer incidence reached its nadir in week 14, after which it started to increase again from week 17 (Fig. 1). Fewer patients (150 DCIS and 1000 invasive tumors) were diagnosed after week 9 in 2020 compared with 2018/2019. At the hospital level, the mean decrease in breast cancer diagnoses during weeks 9–17 was 33.5% in 2020, ranging from an increase of 32.1% to a decrease of 87.3%. Within the 50–74 year age group, the incidence of screen-detected tumors started to drop from week 12 onwards, when screening was temporarily halted, and reached almost zero in week 14. The incidence of non-screen-detected tumors dropped from week 11 onwards, reaching its nadir in week 14, after which it increased again in week 17 (Fig. 2).

**Fig. 3:**
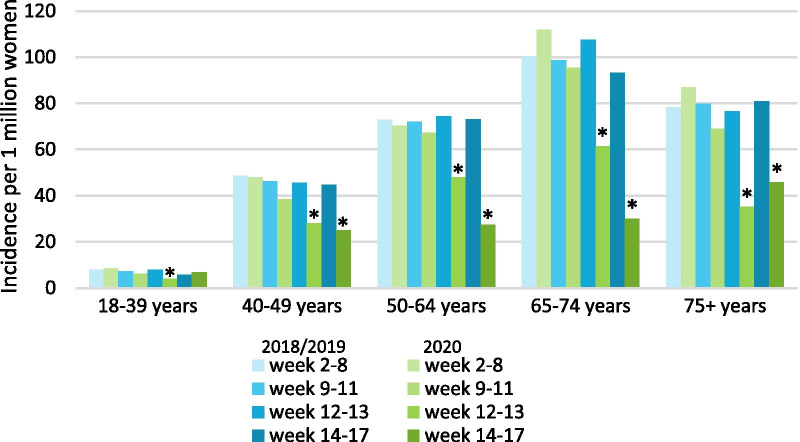
Average weekly incidence, per 1 million women of that specific age category, stratified by period. Incidence is expressed per 1 million women of that specific age category living in the Netherlands at the start of the year. *The incidence in that period is significantly lower compared to the average incidence in the same period of 2018/2019 (*p* < 0.05)

**Fig. 4:**
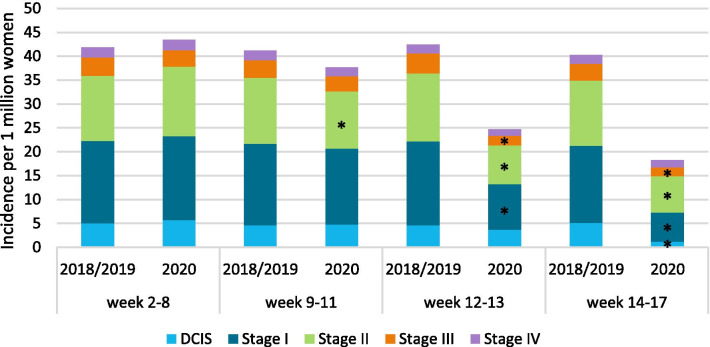
Average weekly incidence of different stage tumors, per 1 million women, stratified by period. Incidence is expressed per 1 million women living in the Netherlands at the start of the year. *The incidence in that period is significantly lower compared to the average incidence in the same period of 2018/2019 (*p* < 0.05)

**Fig. 5:**
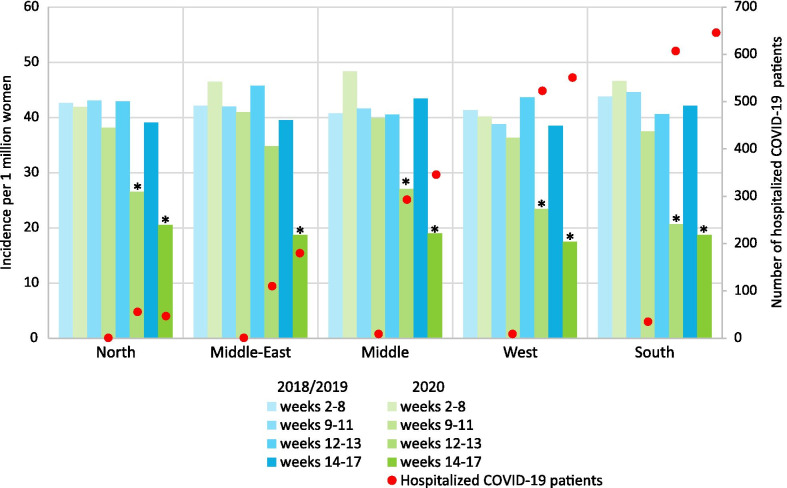
Average weekly incidence and average weekly number of hospitalized COVID-19 patients per region and period. Breast cancer incidence is expressed per 1 million women living in that specific region of the Netherlands at the start of the year. *The incidence in that period is significantly lower compared to the average incidence in the same period of 2018/2019 (*p* < 0.05)

Compared with the same periods in 2018/2019, breast cancer incidence fell significantly in all age categories in period C and D, except in patients younger than 40 years (Fig. 3). In period B, the incidence of stage II tumors fell significantly, while in period C, the incidence of stage I, II, and III tumors fell significantly. The incidence of all tumors, except stage IV, also fell significantly in period D (Fig. 4). Finally, breast cancer incidence fell significantly in all regions except the middle east of the Netherlands in period C and fell significantly in all regions in period D (Fig. 5).

### Treatment

Treatment differences are summarized in Table 2. Compared with 2018/2019, the proportions diagnosed with DCIS in periods A and B of 2020 and not treated within 3 months of diagnosis increased by 7.3% and 8.6%, respectively. The proportion of stage II tumors in period B receiving chemotherapy within 3 months fell by 10.0%. By contrast, the proportional use of hormonal therapy increased by 19.2% for stage IV tumors in period C and by 5.6% for stage I tumors in period D; use also increased among patients aged > 74 years by 23.9% in period C (Additional file 1: Table 3). Concerning BCS, the proportion of stage I tumors treated in period D fell by 14.9%. Finally, the proportion of patients aged 50–64 years in period D who received chemotherapy increased by 16.0%.

**Table 2:** Women per week by initial treatment within 3 months of diagnosis, diagnosis period, and stage

	Total	Untreated	BCS	Mx with IBR	Mx without IBR	Chemotherapy	Hormonal treatment	Other
DCIS								
2018/2019, wk 2–17	42.8	3.9 (9.1)	27.9 (65.2)	5.0 (11.8)	5.4 (12.6)	0.2 (0.4)	0.4 (0.9)	0.0 (0.2)
2020, wk 2–8	49.7	8.1 (16.4) ↑	29.7 (59.8)	5.1 (10.3)	6.4 (12.9)	0.1 (0.3)	0.1 (0.3)	0.0 (0.0)
2020, wk 9–11	41.3	7.3 (17.7) ↑	22.7 (54.8)	3.7 (8.9)	7.0 (16.9)	0.0 (0.0)	0.7 (1.6)	0.0 (0.0)
2020, wk 12–13	32.0	3.5 (10.9)	19.5 (60.9)^a^	2.0 (6.3)	5.5 (17.2)	0.0 (0.0)	1.5 (4.7)^b^	0.0 (0.0)
2020, wk 14–17	9.8	1.0 (10.3)	4.5 (46.2)	2.0 (20.5)	1.8 (17.9)	0.3 (2.6)	0.3 (2.6)	0.0 (0.0)
Stage I								
2018/2019, wk 2–17	147.0	2.7 (1.8)	103.0 (70.1)	10.1 (6.9)	16.8 (11.4)	7.1 (4.8)	7.3 (4.9)	0.2 (0.1)
2020, wk 2–8	153.1	2.6 (1.7)	104.6 (68.3)	8.9 (5.8)^c^	18.0 (11.8)	9.1 (6.0)	9.9 (6.4)	0.1 (0.1)
2020, wk 9–11	138.7	1.7 (1.2)	95.0 (68.5)	10.0 (7.2)	17.7 (12.7)	2.3 (1.7) ↓	12.0 (8.7)^d^	0.0 (0.0)
2020, wk 12–13	83.5	1.0 (1.2)	55.5 (66.5)	3.5 (4.2)	9.5 (11.4)	3.0 (3.6)	11.0 (13.2)^e^	0.0 (0.0)
2020, wk 14–17	52.5	0.8 (1.4)	29.0 (55.2) ↓	4.8 (9.0)	7.5 (14.3)	5.0 (9.5) ↑	5.5 (10.5) ↑	0.0 (0.0)
Stage II								
2018/2019, wk 2–17	119.1	2.6 (2.2)	36.3 (30.5)	5.3 (4.5)	20.9 (17.6)	37.5 (31.5)	16.0 (13.4)	0.4 (0.4)
2020, wk 2–8	127.1	2.9 (2.2)	35.9 (28.2)	5.9 (4.6)	21.7 (17.1)	38.7 (30.4)	21.9 (17.2)^f^	0.3 (0.2)
2020, wk 9–11	105.3	3.3 (3.2)	32.0 (30.4)	3.3 (3.2)	20.0 (19.0)	22.7 (21.5) ↓	24.0 (22.8)^d^	0.0 (0.0)
2020, wk 12–13	71.0	1.0 (1.4)	22.5 (31.7)	1.5 (2.1)	10.5 (14.8)	20.0 (28.2)	15.5 (21.8)^g^	0.0 (0.0)
2020, wk 14–17	66.0	1.0 (1.5)	17.3 (26.1)	2.0 (3.0)	12.3 (18.6)	22.5 (34.1)	11.0 (16.7)	0.0 (0.0)
Stage III								
2018/2019, wk 2–17	32.7	0.8 (2.6)	2.0 (6.2)	0.6 (1.8)	5.9 (18.1)	17.5 (53.6)	5.3 (16.2)	0.5 (1.4)
2020, wk 2–8	29.9	1.1 (3.8)	2.4 (8.1)	0.4 (1.4)	6.0 (20.1)	14.4 (48.3)	5.4 (18.2)	0.0 (0.0)
2020, wk 9–11	27.3	0.3 (1.2)	1.3 (4.9)	0.3 (1.2)	5.3 (19.5)	14.0 (51.2)^h^	6.0 (22.0)	0.0 (0.0)
2020, wk 12–13	17.5	0.0 (0.0)	0.5 (2.9)	1.5 (8.6) ↑	2.5 (14.3)	6.0 (34.3)	7.0 (40.0)^i^	0.0 (0.0)
2020, wk 14–17	15.5	0.3 (1.6)	0.8 (4.8)	1.0 (6.5) ↑	2.3 (14.5)	9.3 (59.7)	2.0 (12.9)	0.0 (0.0)
Stage IV								
2018/2019, wk 2–17	17.2	1.6 (9.3)	0.3 (2.0)	0.0 (0.2)	0.6 (3.5)	5.7 (33.3)	7.4 (43.3)	1.5 (8.5)
2020, wk 2–8	19.1	3.1 (16.4) ↑	0.4 (2.2)	0.0 (0.0)	0.9 (4.5)	6.3 (32.8)	7.4 (38.8)	1.0 (5.2)
2020, wk 9–11	16.3	2.0 (12.2)	0.7 (4.1)	0.0 (0.0)	0.7 (4.1)	5.3 (32.7)^j^	7.7 (46.9)	0.0 (0.0)
2020, wk 12–13	12.0	1.0 (8.3)	0.0 (0.0)	0.0 (0.0)	0.0 (0.0)	3.5 (29.2)^k^	7.5 (62.5) ↑	0.0 (0.0)
2020, wk 14–17	13.5	1.5 (11.1)	0.0 (0.0)	0.0 (0.0)	0.3 (1.9)	5.8 (42.6)^l^	5.3 (38.9)^m^	0.8 (5.6)

Data are shown as average n (%). We compared data for weeks 2–17, 2020 (the COVID-19 period) with those for weeks 2–17, 2018/2019 using the Mantel–Haenszel test, adjusted by age (< 40, 40–50, 50–65, 65–74, > 74 years)

The arrows show statistically significant data corrected for age: ↑ = more patients received this therapy; ↓ = fewer patients received this therapy

BCS, breast conserving surgery; DCIS, ductal carcinoma in situ; IBR, immediate breast reconstruction; Mx, mastectomy; wk, week

Analyses stratified per age group was performed and significant differences are explained:

^a^Lower proportion of patients 65–74 years old with DCIS received BCS

^b^Higher proportion of patients 65–74 years old with DCIS received hormonal treatment

^c^Higher proportion of patients < 40 years old with stage I received mastectomy with IBR

^d^Higher proportion of patients 50–74 years old with stage I or stage II received hormonal treatment

^e^Higher proportion of patients < 75 years old with stage I received hormonal treatment

^f^Higher proportion of patients 40–74 years old with stage II received hormonal treatment

^g^Higher proportion of patients < 50 years old or > 74 years old with stage II received hormonal treatment

^h^Higher proportion of patients < 40 years old with stage III received chemotherapy

^i^Higher proportion of patients < 50 years old or 65–74 years old with stage III received hormonal treatment

^j^Higher proportion of patients < 40 years old with stage IV received chemotherapy

^k^Higher proportion of patients 40–49 years old with stage IV received chemotherapy

^l^Higher proportion of patients aged < 40 years old or 50–64 years old with stage IV received chemotherapy

^m^Higher proportion of patients 40–49 years old or 65–74 years old with stage IV received hormonal treatment

After adjusting for age group, SES, and region, there was a greater odds of DCIS diagnosed in period A or B not being treated within 3 months of diagnosis compared with the reference period (Table 3). In addition, after adjusting for age group, SES, subtype, stage and region, patients diagnosed with an invasive tumor in period A, B, or, C of 2020 were more likely to receive hormonal treatment initially. The odds of receiving chemotherapy were lower for patients diagnosed in period B or C, while the odds of undergoing mastectomy with IBR was lower in period C. In period D, the odds of undergoing BCS were lower and the odds of receiving chemotherapy were higher.

**Table 3:** Association between diagnosis period and type of initial treatment

	Not yet treated	BCS	Mx with IBR	Mx without IBR	Chemotherapy	Hormonal treatment	Other
DCIS^a^							
Weeks 2–8	2.04 (1.44–2.88)	0.81 (0.63–1.04)	0.87 (0.58–1.32)	0.95 (0.66–1.36)	0.71 (0.08–6.32)	0.27 (0.03–2.19)	NA
Weeks 9–11	2.18 (1.31–3.61)	0.72 (0.49–1.05)	0.56 (0.28–1.10)	1.36 (0.82–2.26)	NA	2.13 (0.40–11.33)	NA
Weeks 12–13	1.38 (0.61–3.13)	0.80 (0.47–1.36)	0.44 (0.15–1.29)	1.38 (0.70–2.72)	NA	18.32 (3.68–91.30)	NA
Weeks 14–17	1.07 (0.37–3.11)	0.53 (0.28–1.04)	1.92 (0.78–4.74)	1.40 (0.60–3.25)	5.98 (0.57–62.65)	2.13 (0.22–20.53)	NA
Invasive^b^							
Weeks 2–8	1.11 (0.84–1.48)	0.93 (0.83–1.04)	0.93 (0.74–1.16)	1.01 (0.88–1.16)	0.96 (0.82–1.12)	1.23 (1.05–1.44)	0.50 (0.25–0.98)
Weeks 9–11	0.95 (0.60–1.50)	0.93 (0.79–1.10)	0.97 (0.70–1.36)	1.11 (0.91–1.36)	0.59 (0.45–0.76)	1.92 (1.53–2.41)	NA
Weeks 12–13	0.63 (0.27–1.45)	0.91 (0.70–1.17)	0.52 (0.28–0.96)	0.90 (0.65–1.24)	0.66 (0.45–0.95)	3.01 (2.20–4.11)	NA
Weeks 14–17	0.83 (0.48–1.45)	0.75 (0.61–0.92)	1.08 (0.73–1.59)	1.11 (0.87–1.41)	1.31 (1.01–1.70)	1.26 (0.96–1.65)	0.42 (0.13–1.38)

Logistic regression calculating the odds ratios and 95% confidence intervals to investigate the association between period of diagnosis of the tumor (week 2–17 2018/2019 as a reference) and type of initial treatment, for DCIS and invasive tumors separately

BCS, breast conserving surgery; DCIS, ductal carcinoma in situ; IBR, immediate breast reconstruction; Mx, mastectomy; NA, not applicable

^a^Odds ratios are adjusted for age groups, socioeconomic status, and region

^b^Odds ratios are adjusted for age groups, socioeconomic status, stage, subtype, and region

There was a longer interval between diagnosis and initial treatment in period A compared with the reference period for patients with invasive tumors receiving BCS, mastectomy without IBR, or hormonal treatment (Additional file 1: Table 4). By contrast, shorter intervals were seen for those in period B receiving either mastectomy with IBR or hormonal treatment, as well as for those in period C receiving BCS, mastectomy with/without IBR, or hormonal treatment. The median time from diagnosis to initial treatment in the reference period was 27 days (interquartile range (IQR): 20–36) compared with 29 days (IQR: 21–40; *p* < 0.001) in period A, 22 days (IQR: 14–31, *p* < 0.001) in period C, and 25 days (IQR: 18–35, *p* < 0.001) in period D.

## Discussion

As expected, we found that the incidence of breast cancer started to decline after the social lockdown and the temporary pause in screening at week 12. This decrease was seen in all age groups and all regions, and it was unrelated to the severity of the COVID-19 outbreak based on the number of patients hospitalized with COVID-19. The incidence of stage IV tumors did not decline. There was also a clear change in initial treatment received by patients diagnosed during the outbreak period, with fewer undergoing either BCS or mastectomy plus IBR, and more receiving primary hormonal treatment. Moreover, the interval between diagnosis and initial treatment decreased compared with 2018/2019.

The fall in breast cancer incidence was greatest among patients aged 50–74 years, consistent with those eligible for screening, when screening programs were temporarily halted from week 12. The increased reluctance of patients to visit their GP and the lack of capacity at GPs was reflected by the decrease in incidence across all age groups. Similarly, the decline in incidence among patients aged ≥ 75 years in weeks 12–13 may have reflected the advice that the vulnerable (e.g., the elderly and people with chronic diseases), stay at home.

Given that screening detects most DCIS (72%) and stage I tumors (64%) [8], we anticipated that the temporary cessation of screening would cause these stages to have the greatest decrease in incidence. Patients with suspicious lesions detected at screening are normally referred to hospital within 2 weeks, meaning that weeks 12–13 still reflected patients with DCIS referred from screening, and explaining the delayed decrease from week 14. The incidence of stage II tumors had already started to decline in weeks 9–11, but unlike the early stage tumors, only 38% of these are typically detected by screening. In this instance, the earlier decline in incidence may reflect reluctance to visit a GP despite symptoms/complaints or a lack of capacity at GPs. By contrast, given that only 9% of stage IV tumors are detected by screening and present with worrisome symptoms that are unlikely to be ignored, it was unsurprising that their incidence did not decrease.

Despite the north of the Netherlands being least affected by COVID-19, we found no regional differences in diagnosis rates or treatment strategies between regions. This reflects the national impact of the measures. The increase in incidence in week 17 likely reflects the national call by official authorities in weeks 14 and 15 to visit a GP when experiencing symptoms. The national screening program restart only began in mid-June and could not have influenced the incidence.

Specific Dutch recommendations mentioned that treatment can be delayed for DCIS (Additional file 1: Table 1). The COVID-19 Pandemic Breast Cancer Consortium (CPBCC) also recommended delaying surgery for patients with DCIS, while the European Society for Medical Oncology (ESMO) gave the lowest priority to surgery for DCIS (except for high-grade DCIS) [9, 10]. Our results reflect the implementation of these recommendations. Although research has shown that delaying surgery for up to 60 days did not negatively affect disease-free survival or nodal status in patients with DCIS [11], other research has shown that a greater delay to surgery is associated with a greater risk of finding invasive cancer [12]. It has also been reported that 21% of patients diagnosed clinically with DCIS appear to have an invasive tumor at surgery [13]. Hence, delaying surgery might not be appropriate for all patients. Our research also showed that patients diagnosed with an invasive tumor before the pandemic may have experienced a delay in treatment as hospitals transitioned to accommodate the new health care demands. Patients diagnosed with an invasive tumor during the COVID-19 pandemic did not experience a treatment delay and may have even been treated earlier, indicating successful transition to the new treatment plan. Furthermore, the Dutch, the CPBCC, and the ESMO guidelines recommend using neoadjuvant hormonal treatment for patients with HR-positive tumors [9, 10], which we can confirm was implemented. Combining data from international trials, researchers have reported that hormonal treatment can lead to a safe delay in surgery for at least 6 months in some patients (e.g., postmenopausal, early stage, estrogen receptor-positive, and HER2-negative) [14].

To reduce pressure on operating room capacity and to lower the risk of complications after breast surgery, the Association of Breast Surgery and the American Society of Plastic Surgeons recommended avoiding IBR [15, 16], while ESMO give a low priority to breast reconstructions [10]. Although this was not specified in the Dutch recommendations, we observed a decrease in the use of IBR. Moreover, the ESMO guideline states that chemotherapy can be omitted in patients if the risk-to-benefit appraisal for neoadjuvant chemotherapy is uncertain, only after careful discussion with the patient [17]. The Dutch guidelines did not discourage the use of neoadjuvant chemotherapy, but they did recommend adjusting the doses or types of chemotherapy to prevent neutropenia. The lower use of chemotherapy at the beginning of the pandemic was probably related to limitations placed on hospital visits and the expected increased risk of COVID-19 complications. The decrease in patients hospitalized for COVID-19 after week 17 probably led to the increased use of chemotherapy in patients diagnosed toward the end of the pandemic period that we studied [1]. Hospitals might have decided to treat their patients with chemotherapy because of the lower COVID-19 risk. In the current study, 38 patients were recorded as having clinically suspected COVID-19, and of these, 29% received initial treatment with chemotherapy.

This study, which we believe is the first of its kind, benefited from using data from the NCR for all women diagnosed with breast cancer in the Netherlands, thereby accurately reflecting daily practice. However, the study has some important limitations. First, the use of neoadjuvant chemotherapy has been increasing over time [18, 19], so the decrease in chemotherapy use during the COVID-19 period may be an underestimation. Second, we did not consider hospital transfers when calculating the number of patients hospitalized with COVID-19 in each region, although we have no reason to doubt that our data offer a good representation of the true burden of COVID-19 in each region. Third, the distribution in the number of diagnoses in each age category and region differed in weeks 2–8 between 2020 and 2018/2019, meaning that we cannot exclude the impact of data fluctuations unrelated to the COVID-19 pandemic. As such, these results should be interpreted with care. Finally, we limited our study to the first treatment given within 3 months of diagnosis, which neglects any changes in therapy or delays in adjuvant therapy. Future research will require a longer follow-up period to investigate the effects of the COVID-19 pandemic on the management of patients with breast cancer throughout treatment.

## Conclusion

The COVID-19 outbreak, related societal measures, and cessation of breast cancer screening led to about 1,150 missed cases of breast cancer. However, we found that this reduction mainly occurred for the lowest stage disease, and as such, it is hoped that the delay in diagnosis will not have had a large impact on long-term outcomes. Moreover, patients diagnosed after week 8 experienced no delays in treatment, though it was notable that initial treatments moved away from surgical options throughout (e.g., BCS and mastectomy with IBR) and seemed to be replaced by primary hormonal treatment. Future studies will need to determine the long-term effects of both the delayed diagnosis and the changes in treatment strategies on outcomes. Only then will we be able to make a definitive conclusion. In the meantime, there are three key lessons to be learned from this study: 1) it is important to maintain a functioning national screening program during a pandemic to prevent a major backlog in breast cancer diagnosis; 2) patients, especially those aged ≥ 75 years, should be encouraged to visit a GP when experiencing symptoms; and 3) recommendations can be implemented rapidly nationwide thanks to good communication and management both nationally and locally.

## Supplementary Information

**Additional file 1: Table 1**. Recommendations how to prioritize and adapt treatment for breast cancer patients during the COVID-19 outbreak. List of recommendations formulated by the Scientific associations (Dutch Society of Medical Oncology (NVMO), Dutch Society of Surgical Oncology (NVCO), and Dutch Society of Radiotherapy and Oncology (NVRO) [4]) on how to prioritize and adapt treatment for breast cancer patients during the COVID-19 outbreak. Recommendations were introduced on 22 March 2020. **Table 2**. Baseline characteristics of patients diagnosed in weeks 2–17 2018 or 2019. Comparison between the baseline characteristics of patients diagnosed in weeks 2–17 2018 and those diagnosed in weeks 2–17 2019. **Table 3**. Women per week by initial treatment within 3 months of diagnosis, diagnosis period, and age. We compared data for weeks 2–17 of 2020 (the COVID-19 period) with those for weeks 2–17 of 2018/2019 using the Mantel–Haenszel test, adjusted by stage (DCIS, stage I, stage II, stage III, stage IV). **Table 4**. Association between diagnosis period and days between diagnosis and initial treatment. Cox regression analyses calculating the hazard ratios and 95% confidence intervals to investigate the association between period of diagnosis of the invasive tumor (week 2–17 2018/2019 as a reference) and days between diagnosis and initial treatment, per initial treatment. Hazard ratios are adjusted for age groups, socioeconomic status, subtype, and region.

## Data Availability

All data collected for the study will be made available via the NCR upon request and after approval of a proposal from the date of publication. The plan for the statistical analysis will be made available by the corresponding author upon request.

## Abbreviations

BCS: Breast conserving surgery
CI: Confidence interval
CPBCC: COVID-19 Pandemic Breast Cancer Consortium
DCIS: Ductal carcinoma in situ
ESMO: European Society for Medical Oncology
GP: General practitioner
HER2: Human epidermal growth factor receptor 2
HR: Hormone receptor
IBR: Immediate breast reconstruction
IQR: Interquartile range
NABON: National Breast Cancer Organization of the Netherlands
NCR: Netherlands cancer registry
SES: Socioeconomic status
