# Supplementary material for: Impact of the COVID-19 pandemic on diagnosis, stage, and initial treatment of breast cancer in the Netherlands: a population-based study
Source: J Hematol Oncol. 2021 Apr 17;14:64. doi: 10.1186/s13045-021-01073-7 (PMC8052935; doi:10.1186/s13045-021-01073-7)
Supplement: Supplementary file 1 — Additional file 1: Table 1. Recommendations how to prioritize and adapt treatment for breast cancer patients during the COVID-19 outbreak. List of recommendations formulated by the Scientific associations (Dutch Society of Medical Oncology (NVMO), Dutch Society of Surgical Oncology (NVCO), and Dutch Society of Radiotherapy and Oncology (NVRO) [4]) on how to prioritize and adapt treatment for breast cancer patients during the COVID-19 outbreak. Recommendations were introduced on 22 March 2020. Table 2. Baseline characteristics of patients diagnosed in weeks 2–17 2018 or 2019. Comparison between the baseline characteristics of patients diagnosed in weeks 2–17 2018 and those diagnosed in weeks 2–17 2019. Table 3. Women per week by initial treatment within 3 months of diagnosis, diagnosis period, and age. We compared data for weeks 2–17 of 2020 (the COVID-19 period) with those for weeks 2–17 of 2018/2019 using the Mantel–Haenszel test, adjusted by stage (DCIS, stage I, stage II, stage III, stage IV). Table 4. Association between diagnosis period and days between diagnosis and initial treatment. Cox regression analyses calculating the hazard ratios and 95% confidence intervals to investigate the association between period of diagnosis of the invasive tumor (week 2–17 2018/2019 as a reference) and days between diagnosis and initial treatment, per initial treatment. Hazard ratios are adjusted for age groups, socioeconomic status, subtype, and region. [file 13045_2021_1073_MOESM1_ESM.docx]

**Additional file 1**

**Supplementary Table 1** Recommendations how to prioritize and adapt treatment for breast cancer patients during the COVID-19 outbreak

| **No delay** | **Limited delay** |
| --- | --- |
| Surgical intervention:   - - Progressive disease during NAC   - TNBC without positive LN   - Locally advanced tumor in need of primary surgical treatment   Medical oncological interventions:   - - Neoadjuvant chemotherapy:     - TNBC with positive LN     - HER2+ tumors, unless stage T1N0   - Adjuvant chemotherapy:     - TNBC | Surgical intervention:   - - Patients with HR+ tumors who receive NAC and can tolerate hormonal treatment   - Patients with HER2+ tumor which respond to NAC and in whom trastuzumab is being continued   Medical oncological interventions:   - - Adjuvant chemotherapy:     - HR+ tumor     - HER2+ tumor <3cm, adapt the treatment schedule   Treatment as part of a study: Continue treatment, unless the study is set on hold   - Paclitaxel + trastuzumab adjuvant |
| **Optional delay** | **Delay** |
| Surgical intervention:   - - Patients with HR+ tumors who can tolerate hormonal treatment   - DCIS   - Diagnostic surgery   Medical oncological interventions:   - - Palliative chemotherapy     - HR+ tumor     - TNBC     - HER2+ tumor | Surgical intervention:   - - Preventive surgery   - Benign tumor   - Tumor responding to neoadjuvant hormonal treatment |

DCIS, ductal carcinoma in situ; HR, hormone receptor; LN, lymph node; NAC, neoadjuvant chemotherapy; TNBC, triple negative breast cancer.

Recommendations were introduced on 22 March 2020 and set up by the Scientific associations: Dutch Society of Medical Oncology (NVMO) and the Dutch Society of Surgical Oncology (NVCO)^1^.

**Supplementary Table 2** Baseline characteristics of patients diagnosed in weeks 2–17 2018 or 2019

|  |  | **Weeks 2-17 2018** | **Weeks 2-17 2019** | **P** |
| --- | --- | --- | --- | --- |
| **Patients** |  | 5685 | 5838 |  |
| **Week of diagnosis** | 2-8 | 2542 (44.7) | 2553 (43.7) | 0.18 |
|  | 9-11 | 1022 (18.0) | 1127 (19.3) |  |
|  | 12-13 | 750 (13.2) | 725 (12.4) |  |
|  | 14-17 | 1371 (24.1) | 1433 (24.6) |  |
| **Age** | <40 | 270 (4.8) | 272 (4.7) | 0.94 |
|  | 40-50 | 853 (15.0) | 861 (14.8) |  |
|  | 50-65 | 2075 (36.5) | 2107 (36.1) |  |
|  | 65-75 | 1484 (26.1) | 1562 (26.8) |  |
|  | >75 | 1003 (17.6) | 1036 (17.8) |  |
| **SES** | High | 1725 (30.3) | 1675 (28.7) | 0.08 |
|  | Medium | 2183 (38.4) | 2348 (40.2) |  |
|  | Low | 1751 (30.8) | 1792 (30.7) |  |
|  | Unknown | 26 (0.5) | 23 (0.4) |  |
| **Morphology** | DCIS | 667 (11.7) | 705 (12.1) | 0.61 |
|  | Invasive ductal | 3939 (69.3) | 4007 (68.6) |  |
|  | Invasive lobular | 769 (13.5) | 826 (14.2) |  |
|  | Other | 310 (5.5) | 300 (5.1) |  |
| **Stage** | DCIS | 667 (11.7) | 705 (12.1) | 0.13 |
|  | Stage I | 2387 (42.0) | 2325 (39.8) |  |
|  | Stage II | 1844 (32.4) | 1970 (33.7) |  |
|  | Stage III | 496 (8.7) | 553 (9.5) |  |
|  | Stage IV | 280 (4.9) | 275 (4.7) |  |
| **Subtype** | HR+/HER2+ | 436 (7.7) | 453 (7.8) | 0.77 |
|  | HR+/HER2- | 3777 (66.4) | 3876 (66.4) |  |
|  | HR-/HER2+ | 187 (3.3) | 206 (3.5) |  |
|  | HR-/HER2- | 573 (10.1) | 553 (9.5) |  |
|  | Unknown | 712 (12.5) | 750 (12.9) |  |
| **Region** | North | 564 (9.9) | 592 (10.1) | 0.27 |
|  | Middle-East | 505 (8.9) | 543 (9.3) |  |
|  | Middle | 1085 (19.1) | 1192 (20.4) |  |
|  | West | 2262 (39.8) | 2256 (38.6) |  |
|  | South | 1269 (22.3) | 1254 (21.5) |  |
|  | Unknown | 0 (0.0) | 1 (0.0) |  |
| **Screened** | Yes | 1893 (33.3) | 1860 (31.9) | 0.14 |
|  | No | 1568 (27.6) | 1695 (29.0) |  |
|  | Not eligible for screening | 2126 (37.4) | 2169 (37.2) |  |
|  | Unknown | 98 (1.7) | 114 (2.0) |  |
| DCIS, ductal carcinoma in situ; HER2, human epidermal growth factor receptor 2; HR, hormone receptor; SES, socioeconomic status.  Data are reported as N(%) of patients diagnosed in weeks 2–17 of 2018 or 2019.  The p-value was calculated on known values only, using the chi-square test to compare patients diagnosed in 2018 with those diagnosed in 2019. | | | | |

**Supplementary Table 3** Women per week by initial treatment within 3 months of diagnosis, diagnosis period, and age

|  | **Total** | **Not yet treated** | **BCS** | **Mx with IBR** | **Mx without IBR** | **Chemotherapy** | **Hormonal treatment** | **Other** |
| --- | --- | --- | --- | --- | --- | --- | --- | --- |
| **< 40** |  |  |  |  |  |  |  |  |
| 2018/2019, wk 2–17 | 16.9 | 0.3 (1.7) | 3.1 (18.5) | 2.0 (12.0) | 1.1 (6.3) | 9.5 (56.2) | 0.7 (3.9) | 0.3 (1.5) |
| 2020, wk 2–8 | 20.1 | 0.6 (2.8) | 2.4 (12.1) | 3.9 (19.1) ↑ | 1.0 (5.0) | 10.3 (51.1) | 1.7 (8.5) ↑ | 0.3 (1.4) |
| 2020, wk 9–11 | 14.7 | 0.3 (2.3) | 2.7 (18.2) | 2.0 (13.6) | 2.3 (15.9) ↑ | 7.0 (47.7)^a^ | 0.3 (2.3) | 0.0 (0.0) |
| 2020, wk 12–13 | 9.5 | 0.0 (0.0) | 1.5 (15.8) | 1.5 (15.8) | 1.0 (10.5) | 3.0 (31.6) | 2.5 (26.3) ↑ | 0.0 (0.0) |
| 2020, wk 14–17 | 15.3 | 0.0 (0.0) | 2.0 (13.1) | 2.3 (14.8) | 0.5 (3.3) | 10.0 (65.6) | 0.5 (3.3) | 0.0 (0.0) |
| **40–49** |  |  |  |  |  |  |  |  |
| 2018/2019, wk 2–17 | 53.4 | 1.5 (2.8) | 18.8 (35.1) | 6.3 (11.9) | 4.2 (7.8) | 20.9 (39.1) | 1.6 (2.9) | 0.2 (0.4) |
| 2020, wk 2–8 | 53.0 | 1.3 (2.4) | 18.9 (35.6) | 4.7 (8.9) | 5.3 (10.0) | 20.1 (38.0) | 2.6 (4.9) | 0.1 (0.3) |
| 2020, wk 9–11 | 42.3 | 2.3 (5.5) | 14.7 (34.6) | 5.7 (13.4) | 4.7 (11.0) | 12.3 (29.1) | 2.7 (6.3) ↑ | 0.0 (0.0) |
| 2020, wk 12–13 | 31.0 | 0.5 (1.6) | 10.5 (33.9) | 3.5 (11.3) | 3.0 (9.7) | 9.5 (30.6)^b^ | 4.0 (12.9) ↑ | 0.0 (0.0) |
| 2020, wk 14–17 | 27.0 | 0.5 (1.9) | 9.5 (35.2) | 3.5 (13.0) | 2.0 (7.4) | 10.3 (38.0) | 1.3 (4.6) | 0.0 (0.0) |
| **50–64** |  |  |  |  |  |  |  |  |
| 2018/2019, wk 2–17 | 130.5 | 4.0 (3.1) | 70.8 (54.3) | 10.4 (8.0) | 12.9 (9.9) | 26.4 (20.2) | 5.3 (4.1) | 0.7 (0.5) |
| 2020, wk 2–8 | 127.6 | 5.3 (4.1) | 63.0 (49.4) ↓ | 9.0 (7.1) | 13.6 (10.6) | 28.4 (22.3) | 8.1 (6.4) ↑ | 0.1 (0.1) |
| 2020, wk 9–11 | 122.3 | 4.7 (3.8) | 63.3 (51.8) | 7.7 (6.3) | 14.7 (12.0) | 17.3 (14.2) ↓ | 14.7 (12.0)^d^ | 0.0 (0.0) |
| 2020, wk 12–13 | 86.5 | 1.5 (1.7) | 52.5 (60.7) | 2.5 (2.9) ↓ | 7.0 (8.1) | 15.5 (17.9) | 7.5 (8.7)↑ | 0.0 (0.0) |
| 2020, wk 14–17 | 49.0 | 1.8 (3.6) | 16.3 (33.2) ↓ | 3.8 (7.7)^c^ | 5.5 (11.2) | 17.8 (36.2) ↑ | 3.8 (7.7) | 0.3 (0.5) |
| **65–74** |  |  |  |  |  |  |  |  |
| 2018/2019, wk 2–17 | 95.0 | 1.8 (1.9) | 58.3 (61.3) | 2.3 (2.4) | 15.8 (16.7) | 9.8 (10.3) | 6.4 (6.7) | 0.6 (0.7) |
| 2020, wk 2–8 | 109.9 | 5.4 (4.9) ↑ | 67.6 (61.5) | 2.6 (2.3) | 15.0 (13.7) | 10.9 (9.9) | 8.3 (7.5) | 0.1 (0.1) |
| 2020, wk 9–11 | 93.7 | 3.0 (3.2) | 55.7 (59.4) | 2.0 (2.1) | 15.3 (16.4) | 7.0 (7.5) | 10.7 (11.4) ↑ | 0.0 (0.0) |
| 2020, wk 12–13 | 60.5 | 3.0 (5.0) | 29.5 (48.8) ↓ | 0.5 (0.8)^e^ | 11.5 (19.0) | 5.0 (8.3) | 11.0 (18.2)^g^ | 0.0 (0.0) |
| 2020, wk 14–17 | 29.3 | 0.0 (0.0) | 13.3 (45.3) ↓ | 0.3 (0.9) | 6.3 (21.4)^f^ | 4.0 (13.7) | 5.3 (17.9) ↑ | 0.3 (0.9) |
| **>74** |  |  |  |  |  |  |  |  |
| 2018/2019, wk 2–17 | 63.6 | 4.2 (6.6) | 18.7 (29.4) | 0.2 (0.3) | 15.7 (24.6) | 1.5 (2.1) | 22.5 (35.4) | 0.8 (1.6) |
| 2020, wk 2–8 | 72.6 | 5.7 (7.9) | 22.0 (30.3) | 0.1 (0.2) | 18.6 (25.6) | 0.6 (0.6) ↓ | 24.9 (34.3) | 0.7 (1.2) |
| 2020, wk 9–11 | 57.7 | 4.3 (7.5) | 16.0 (27.7) | 0.0 (0.0) | 13.7 (23.7) | 1.7 (2.3) | 22.0 (38.2) | 0.0 (0.6) |
| 2020, wk 12–13 | 29.5 | 1.0 (3.4) | 5.0 (16.9) | 0.5 (1.7) | 5.5 (18.6) | 0.0 (0.0) | 17.5 (59.3) ↑ | 0.0 (0.0) |
| 2020, wk 14–17 | 38.0 | 2.3 (5.9) | 11.3 (29.6) | 0.0 (0.0) | 10.0 (26.3) | 1.0 (2.6) | 13.3 (34.9) | 0.3 (0.7) |
| BCS, breast conserving surgery; DCIS, ductal carcinoma in situ; IBR, immediate breast reconstruction; Mx, mastectomy; wk, week.  Data are shown as average n (%). We compared data for weeks 2–17, 2020 (the COVID-19 period) with those for weeks 2–17, 2018/2019 using the Mantel–Haenszel test, adjusted by stage (DCIS, stage I, stage II, stage III, stage IV).  The arrows show statistically significant data corrected for stage: ↑ = more patients received this therapy; ↓ = fewer patients received this therapy.  Analyses stratified per stage group was performed and significant differences are explained:  a: higher proportion of patients < 40 years old with a stage III or IV tumor received chemotherapy  b: higher proportion of patients 40–49 years old with stage IV received chemotherapy  c: higher proportion of patients 50–64 years old with DCIS received mastectomy with IBR  d: higher proportion of patients 50–64 years old with a stage I or II tumor received hormonal treatment  e: higher proportion of patients 65–74 years old with a stage III tumor received mastectomy with IBR  f: higher proportion of patients 65–74 years old with DCIS received mastectomy without IBR  g: higher proportion of patients 65–74 years old with a DCIS or a stage I, III, or IV tumor received hormonal treatment | | | | | | | | |

**Supplementary Table 4** Association between diagnosis period and days between diagnosis and initial treatment

|  | **BCS** | **Mx with IBR** | **Mx without IBR** | **Chemotherapy** | **Hormonal treatment** | **Other** |
| --- | --- | --- | --- | --- | --- | --- |
| Weeks 2–8 | 0.88 (0.82–0.94) | 0.99 (0.80–1.23) | 0.87 (0.77–0.99) | 0.98 (0.88–1.08) | 0.76 (0.67–0.86) | 1.16 (0.45–2.98) |
| Weeks 9–11 | 0.92 (0.83–1.02) | 1.50 (1.08–2.08) | 1.12 (0.94–1.35) | 1.07 (0.90–1.27) | 1.28 (1.07–1.52) | 1.90 (0.20–17.85) |
| Weeks 12–13 | 1.32 (1.12–1.55) | 3.90 (2.10–7.23) | 1.56 (1.15–2.12) | 1.09 (0.85–1.40) | 1.78 (1.41–2.24) | NA |
| Weeks 14–17 | 1.10 (0.95–1.28) | 1.20 (0.83–1.74) | 1.05 (0.85–1.31) | 1.05 (0.89–1.23) | 1.15 (0.93–1.43) | NA |
| BCS, breast conserving surgery; IBR, immediate breast reconstruction; Mx, mastectomy; NA, not applicable.  Cox regression analyses calculating the hazard ratios and 95% confidence intervals to investigate the association between period of diagnosis of the invasive tumor (week 2–17 2018/2019 as a reference) and days between diagnosis and initial treatment, per initial treatment. Hazard ratios are adjusted for age groups, socioeconomic status, subtype, and region.  Hazard ratio < 1: time between diagnosis and initial treatment (event) is longer compared to 2018/2019  Hazard ratio >1: time between diagnosis and initial treatment (event) is shorter compared to 2018/2019 | | | | | | |

**Reference:**

1. Civil YA, van Iersel T, Menke-van der Houven CW, Barbé E, van der Velde S. Prioritering van borstkankerzorg tijdens coronacrisis. *Nederlands Tijdschrift voor Geneeskunde*. 2020;**164**(30):D5123.
